# Genetic consistency between gait analysis by accelerometry and evaluation scores at breeding shows for the selection of jumping competition horses

**DOI:** 10.1371/journal.pone.0244064

**Published:** 2020-12-16

**Authors:** Anne Ricard, Bernard Dumont Saint Priest, Marjorie Chassier, Margot Sabbagh, Sophie Danvy

**Affiliations:** 1 Génétique Animale et Biologie Intégrative, Institut national de recherche pour l’agriculture, l’alimentation et l’environnement, AgroParisTech, Université Paris Saclay, Jouy-en-Josas, France; 2 Institut Français du Cheval et de l’Equitation, Pôle Développement, Innovation et Recherche, Exmes, France; Massey University, NEW ZEALAND

## Abstract

The aim was to assess the efficiency of gaits characteristics in improving jumping performance of sport horses and confront accelerometers and judge scores for this purpose. A sample of 1,477 young jumping horses were measured using accelerometers for walk, trot, and canter. Of these, 702 were genotyped with 541,175 SNPs after quality control. Dataset of 26,914 horses scored by judges in breeding shows for gaits and dataset of 142,682 horses that performed in jumping competitions were used. Analysis of accelerometric data defined three principal components from 64% to 89% of variability explained for each gait. Animal mixed models were used to estimate genetic parameters with the inclusion to up 308,105 ancestors for the relationship matrix. Fixed effects for the accelerometric variables included velocity, gender, age, and event. A GWAS was performed on residuals with the fixed effect of each SNP. The GWAS did not reveal other QTLs for gait traits than the one related to the height at withers. The accelerometric principal components were highly heritable for the one linked to stride frequency and dorsoventral displacement at trot (0.53) and canter (0.41) and moderately for the one linked to longitudinal activities (0.33 for trot, 0.19 for canter). Low heritabilities were found for the walk traits. The genetic correlations of the accelerometric principal components with the jumping competition were essentially nil, except for a negative correlation with longitudinal activity at canter (-0.19). The genetic correlation between the judges’ scores and the jumping competition reached 0.45 for canter (0.31 for trot and 0.17 for walk). But these correlations turned negative when the scores were corrected for the known parental breeding value for competition at the time of the judging. In conclusion, gait traits were not helpful to select for jumping performances. Different gaits may be suitable for a good jumping horse.

## Introduction

Gait characteristics have been widely used in sport horse breeding for decades. The aim is both to improve gaits as a direct trait or to use them as a tool for an indirect prediction of success in competition [1]. Specifically, for the discipline of jumping, gaits are not evaluated during the competition, unlike what is practiced in the dressage discipline. Therefore, gait characteristics for jumping represent a double challenge. The traditional qualities of the specific gaits of dressage horses can be rather antagonistic with the ability to jump [2–5]. However, different gait traits than those identified as favorable for dressage could be indirect tools for the selection of jumping horses. Consequently, it would be of considerable interest to determine which characteristics of gaits are genetically correlated, positively or negatively, with jumping ability. Gait characteristics are usually recorded using linear profiling [6–9] or scoring by judges [10–12]. Judge scores or vet scores may have low reliability compared to objective measurements Objective measurements have been developed using kinematic and accelerometric techniques to quantify gait traits in horses. These techniques have mostly been restricted to small sample sizes and used primarily as clinical tests to detect lameness or reflexive training exercises for riders [13]. However, accelerometry is a rapid and non-invasive tool that can be used on a large sample of horses for selection purposes, especially in combination with genomics as leverage to increase the reliability of the predictions.

In this study, gaits were measured by accelerometry in a large sample of young jumping horses during regular competitions as an extra test. Half of them were genotyped. The aim was to calculate heritability and to perform a genome-wide association study of the gait traits measured by accelerometers. We then compared the use of these traits with the traditional use of assessment scores of young horses in the breeding evaluation of horses when the selection goal is the performance in jumping competitions.

## Materials and methods

Research did not used additional manipulation of the horses than the one required in ordinary official jumping competition. Consequently, it was not necessary to refer to an ethics committee. We received permissions from the owners of the horses as well as the show organizers to collect the data.

### Accelerometry, gait and conformation judge scoring, jumping competitions, and genotyping data

During the spring of both 2015 and 2016, 1,477 young sport horses aged four (42%) and five (58%) years were recorded with an Equimetrix^®^ accelerometer [14] at walk, trot, and canter under saddle. These horses were 50% females, 35% geldings, and 15% males. Their breed was 84% Selle Français (SF), while for the remaining 16% (10% foreign sport horses, 2% Anglo-Arabs, and 4% unregistered horses), 39% had at least one SF parent.

Acceleration of the horses was recorded with the Equimetrix^®^ accelerometer device attached to the girth. Data were recorded at competition events for young jumping horses after the competition was over. Riders were asked to perform a quick gait test including the three gaits in an arena with diagonal lines of 60 meters in order to have a sufficiently long measurement time. For trot and canter, two diagonals were measured: one performed at working gait and the other one at medium gait according to the rider’s feeling. The difference between the two gaits was mostly specific to the horse and lead to two different velocities for each gait. Velocity was measured using a chronometer on this diagonal. A sample of 10 seconds for each gait was extracted for the gait measurement calculations. The raw data were the acceleration recorded at 100 Hz per axis in the dorsoventral, longitudinal, and lateral axes of the horse. Five gaits were distinguished: walk (W), working trot, medium trot, working canter, and medium canter. Eight measurements were then calculated:

- velocity (V),
- stride frequency (SF),
- regularity (R),
- symmetry (S, except for canter),
- dorsoventral displacement (DVD),
- dorsoventral power or activity (DVA),
- longitudinal activity (LGA),
- lateral activity (LTA).

The calculation methods are described in [14–16]. Logarithm transformation was applied to normalize the symmetry and the activity measurements (except for dorsoventral at trot) in order to obtain better Gaussian distributions. Thus, the five gaits of each horse were characterized by a total of 38 measurements. Thirty-two percent of the horses were recorded in 2015 and the remainder in 2016. Combination of the locations and the day of the recording resulted in 26 levels of “event” effects. Height at withers was routinely measured for 97% of these horses (mean 166.75 cm, SD 4.16).

Nearly all of these horses (94%) performed in jumping competitions between 2014 and 2019. They spent between one and six years in competition, with an average of 4.0. Analysis of the jumping competition data was performed with the complete dataset of official jumping competitions from 2002 to 2019 since the birth year 1998, including 142,682 horses, and 612,449 annual performances. Horses born abroad and with a potential partial career outside France were excluded from the analysis. Success in competition was measured by the repetition of an annual performance. The annual performance was the logarithm of the annual sum of points distributed according to the rank and the event. Points were awarded for each event based on the height of the obstacles and the technical difficulty (a Grand Prix event has more points than a Table C event for the same height of obstacles). The distribution of points awarded for an event was done on a traditional exponential basis (S1 File). Points for rank were distributed according to normal scores, i.e. expectation of the order statistic of the same rank in a sample of standard normal random variables of the size of the number of starters, and were transformed on an exponential scale (S1 File). All starters in an event earned points. Finally, points earned by the horse in one event were the product of points allocated to the event and point allocated to the rank. The points were summed over one year and log transformed to ensure a normal distribution between horses.

Only twenty nine percent of the horses measured for accelerometry also participated in breeding shows where conformation and gaits were scored. The analysis of the judges’ scores included all of the data recorded by the Stub Book Selle Français (SBSF) during regular breeding shows for young horses (two to three years old) from 2005 to 2018. They involved nine conformation traits and three gait traits judged in hand for the walk and during free presentation for trot and canter. There were 26,914 horses that were recorded once (for 52% of the horses), twice (37% of the horses), and up to a maximum of nine times for the same horse in 1,540 local, regional, and national shows.

Pedigrees were provided by the IFCE (Institut Français du Cheval et de l’Equitation) on behalf of the breeding organizations. According to the different analyses performed (see below), the number of horses in a pedigree that were used varied from 10,907 to 308,105 over four generations. Links between traits (accelerometry, jumping competition, and scores in breeding shows) were mainly provided by the horses that had the different traits measured and sires with progeny that had the different traits measured, as well as all relationships between the horses. There were 410 sires with progeny in the three traits (average progeny size 3.4 for accelerometry, 141.1 for jumping competition, and 41.6 for breeding shows).

Of the horses measured by accelerometry, 702 were genotyped with the Affymetrix Axiom Equine genotyping array that includes 670,806 SNPs. After quality control (minimum allele frequency > 2%, p-value Hardy-Weinberg equilibrium test > 10^−6^, call rate > 90%), 541,175 SNPs were retained.

### Statistical methods

Different models and methods were applied to answer specific questions with the data. All of the mixed model analyses used WOMBAT software [17].

#### Are the accelerometry traits of working and medium gaits (trot & canter) genetically the same?

To answer this question, a bi-trait model between working and medium trot was devised for each of the eight trot measurements and between working and medium canter for the seven canter measurements. The following model was applied for each of the measurements for working gait on the one hand and medium gait on the other hand:

y=Xb+Za+e (1)

where **y** represents the vector of one measurement, for working gait and for medium gait, **b** the vector of fixed effects, which included gender (female, male, or gelding), age (four or five years), velocity (covariate, except for the analysis of velocity itself), event (26 levels), **a** the random polygenic effect (10,907 horses including ancestors over four generations), and **e** the vector of residuals. **X** and **Z** are incidence matrices. A bi-trait analysis was then performed where the variance-covariance matrices of random effects were: *V*(**a**) = **A** ⊗ **G** and *V*(**e**) = **I** ⊗ **R**, with ⊗ the direct product, **A** the relationship matrix from pedigree, **G** the 2 x 2 matrix of genetic variance-covariance between medium and working gait for each trait, **I** the identity matrix, and **R** the 2 x 2 matrix of residual variance-covariance.

#### How can we summarize the main characteristics of accelerometry for the walk, trot, and canter?

To summarize the main characteristics of the 38 measurements representing gaits, we performed a principal component analysis with phenotypes corrected for the identified fixed effects.

We used:

y=Xb+Zu+e, (2)

where **y** represents the vector of measurements for the walk (eight traits), trot (eight traits) or canter (seven traits), **b** the vector of fixed effects with fixed effect of the type of gait (working/medium) for trot and canter, velocity within type of gaits (two covariates, one for each type of gait, except for the analysis of velocity itself), gender (female, male, or gelding), age (four or five years) and event (26 levels), **u** the random effect of the 1,477 horses for the repeated measurements in trot and canter (no effect for walk), and **e** the vector of residuals. **X** and **Z** are incidence matrices. The variance-covariance matrices of random effects were: $V(u)=I\sigma_{u}^{2}$ and $V(e)=I\sigma_{e}^{2}$.

The principal component analysis was performed using the FactoMineR package [18] with as variables the $\hat{u}$ estimates for trot and canter and $y-X\hat{b}$ for walk. In the rest of the study, the eigenvectors of the three first principal components (PC) of each gait (walk, trot, and canter) were used to compute three linear combinations of the measurements for each gait resulting in nine synthetic gait traits.

#### Genome-wide association study

We performed a genome-wide association study (GWAS) for the nine PCs. Scores for each of the three principal components of each gait were computed for each horse using the linear combination given by eigenvectors obtained by the principal component analysis. A mixed model including both the fixed effect of the SNP and the random genetic value of the horse would be the correct model to analyze these scores. The GWAS was performed using the GenABEL package [19]. GenABEL used a slightly simplified model. The GRAMMAR model [20] was a two steps model. It performed only once the estimation of variance components and effects without the SNP effect and then used the residuals of the model to estimate each SNP effect. The power of this method is very closed to the use of the one step full model and avoid in the same way problems due to the genetic structuration [21] of the data. Residuals in case of repeated measurement (for trot and canter) can be replaced by the estimate of the permanent environment effect $\left(\hat{p}\right)$. Then, in the first step, the model used was a multiple trait animal model:

y=Xb+Za+Zp+e, (3)

with **y** representing the vector one of the nine PCs, **b** the vector of fixed effects, which included gender (female, male, or gelding), age (four or five years), velocity (covariate), event (26 levels), **a** the random polygenic effect (10,907 horses including ancestors over four generations), **p** the random permanent environmental effect (for trot and canter repeated two times), and **e** the vector of residuals. **X** and **Z** are incidence matrices. The variance-covariance matrices of random effects: *V*(**a**) = **A** ⊗ **G**, *V*(***p***) = **I** ⊗ **P**, and *V*(**e**) = **I** ⊗ **R**, with ⊗ the direct product, **A** the relationship matrix from pedigree, **G** the 9 x 9 matrix of genetic variance-covariance between each PC trait, **P** the 9 x 9 matrix of genetic variance-covariance between the permanent environmental effect of the PC trait, **I** the identity matrix, and **R** the 9 x 9 matrix of residual variance-covariance. The residual covariances between trot and canter were null by construction: the environmental correlation was considered in the correlation between the permanent environmental effects.

In the second step the GWAS was performed using:

$${\hat{p}}^{\left(3\right)}=1\mu +X\beta +e$$

with ${\hat{p}}^{\left(3\right)}$ the estimated permanent environmental effect from (3), β the fixed effect of the SNP (541,175 analyzes were performed). A supplementary analysis used the height at wither as performance and the residual of the genetic animal model used in the equivalent first step.

#### Genetic analysis of gaits measured by accelerometry and the height at withers

In a second step, we included height at withers in our model. The multivariate analysis of the gaits involved 10 traits: the nine PCs and the height at withers. The model included as covariates the velocity and the height at withers for the gait components, so that the model was a structured equation model [22, 23] and the multiple trait models (3) were applied with a special pattern for residual correlations to avoid over parametrization and to ensure that the environmental correlation between height and gaits was null. The **R** matrix was a 10 x 10 matrix with 1) zero values between gaits because the environmental correlation was considered by the 10x10 **P** matrix of variance-covariance between the permanent environmental effect ***p*** and 2) zero values between height at withers and gait variables to ensure that the environmental correlation between height and gaits was in the regression coefficient 3) non-zero values between the variables for the same gaits as they were computed from the same data.

#### Genetic correlation between accelerometry, jumping performance, and judges’ scores

Due to the amount of jumping data, the analysis was performed repeatedly with three traits: one of the PCs, one of the judges’ scores, and the overall jumping performance. The same model as before was used for the PCs, as in previous section including height. The model for the jumping performance included as fixed effects a combination (leading to 125 levels) of year (from 2002 to 2019) and age effects (from 4 to 10 years old as steps of one year, 11–12 years old, and 13 years old and older) and gender (male: stallions and geldings together, female). The model for the judges’ scores included fixed effects of age (five levels according to age in days: ≤ 2.0 years, 2–2.5 years, 2.5–3 years, 3 years, 3.5 years, and > 3.5 years), year (from 2005 to 2018), local/regional/national type, category (six levels according to age in year and gender), gender (male/female), and show (1,540 levels). There were two random effects: **a** the vector of the genetic additive values and **p** the vector of the permanent environmental effect. The variance matrices were *V*(**a**) = **A** ⊗ **G**, *V*(***p***) = **I** ⊗ **P**, and *V*(**e**) = **I** ⊗ **R**, with ⊗ the direct product, **A** the relationship matrix from the pedigree (308,105 horses), **G** the 3 x 3 matrix of the genetic variance-covariance between the PC trait, the jumping performance, and the judges’ scores, **P** the 3 x 3 matrix of the genetic variance-covariance between the permanent environmental effect of the three traits, **I** the identity matrix, and **R** the 3 x 3 diagonal matrix of the residual variance-covariance.

An additional analysis of the judges’ scores alone was performed to estimate the genetic correlation between them. The model included the already mentioned fixed effects, genetic values, and permanent environmental effects. The residual variance-covariance matrix included the covariance residuals between the scores obtained in the same event. The pedigree restricted to horses measured in breeding shows included 75,528 horses.

#### Genetic correlations between jumping competitions and judges’ scores using estimated breeding values

Estimated Breeding Values (EBV) for the jumping competition trait are available to the public for all horses in France since birth (https://www.ifce.fr/). We calculated the EBV for all horses judged in breeding shows obtained the year of the show, deleting results in competitions obtained after this date from the whole dataset (15 evaluations for last year of performance from 2004 to 2018). These EBV obtained at two or three years of age were, therefore, calculated only from parent information, rather than from their own performances, and are referred to as the parent average (PA) EBV. We then performed a regression with, as the dependent variable, the judges’ scores in breeding shows and, as the independent variable, the PA obtained the year of scoring. Residuals of this regression were used as a new scoring: the trait was the supplemental information provided by the assessment of conformation and gait, knowing the estimated genetic value for jumping competition. This new trait was used in bivariate analysis with the jumping competition performance to compute new genetic correlations, with the same models as in the previous section. In this case, the genetic correlation expected was lower than the initial genetic correlation between the two traits (when positive) and decreased with the increase of PA reliability: $r_{a}^{*}=\frac{r_{a}(1-{CD}_{0})}{\sqrt{\left(1-r_{a}^{2}{CD}_{0}\right)}}$, with *r*_*a*_ the initial genetic correlation and *CD*_0_ the reliability of the parental evaluation used to calculate the residual of scores (S2 File).

## Results

Elementary statistics on the raw variables used to describe the accelerometric measurements of gaits are presented in Table 1. Elementary statistics on the judges’ scores during breeding shows are presented in Table 2.

**Table 1:** Descriptive statistics of the accelerometry variables of the sample of young four or five years old jumping horses (n = 1477) (mean ± SD).

Gait^a^	MT	WT	MC	WC	W
N	1398	1451	1372	1357	1436
Velocity (m/s)	4.35 ± 0.43	3.57 ± 0.43	6.22 ± 0.7	5.20 ± 0.58	1.77 ± 0.2
Stride Frequency (/s)	1.46 ± 0.08	1.37 ± 0.07	1.73 ± 0.08	1.68 ± 0.07	0.91 ± 0.06
regularity	312.0 ± 47.0	348.4 ± 36.2	113.3 ± 24.8	127.2 ± 22.4	178.3 ± 47.3
symmetry	5.45 ± 0.20	5.50 ± 0.20			5.30 ± 0.26
dorsoventral displacement (cm)	10.38 ± 1.89	10.75 ± 1.85	21.36 ± 2.45	20.50 ± 2.14	3.97 ± 1.46
dorsoventral activity (W/kg)	19.45 ± 3.30	14.73 ± 3.19	2.93 ± 0.18	2.70 ± 0.17	-0.22 ± 0.46
longitudinal activity (W/kg)	2.36 ± 0.32	2.14 ± 0.31	2.96 ± 0.46	2.46 ± 0.42	0.96 ± 0.40
latteral activity (W/kg)	1.34 ± 0.33	0.98 ± 0.35	1.95 ± 0.33	1.68 ± 0.31	0.24 ± 0.35

^a^ WT = working trot, MT = Medium Trot, WC = Working canter, MC = medium canter, W = walk.

**Table 2:** Descriptive statistics of the judges’ scores during breeding shows.

Score	N	Mean	SD	Min.	Max.
**Conformation**					
Neck	44,285	13.9	1.8	6	20
Forehand (shoulder, forearm)	38,917	13.9	1.6	6	19
Withers-Back-Loins	44,285	13.9	1.6	6	19
Hindquarters (croup, pelvis, thigh)	43,432	13.8	1.7	6	19
Joints	15,573	14.3	1.3	8	18
Forelimb	44,289	12.9	1.8	6	18
Hindlimb	44,287	13.2	1.7	6	18
Impression	44,323	14.1	1.6	6	19
Chic	36,512	13.9	1.8	6	20
**Gaits**					
Trot	44,102	13.5	1.9	6	20
Canter	43,723	13.5	1.8	6	20
Walk	43,352	13.4	1.7	6	19

### Are the accelerometry traits of working and medium gaits (trot & canter) genetically the same?

Very high genetic correlations between working and medium gait allowed measurements in the two gaits to be considered as the same trait (Tables 3 and 4). An exception was for regularity and symmetry at trot, which behaved quite differently: they showed low heritability (mostly ≤ 0.07) traits, not significantly different from zero and with a very low repeatability (0.08 to 0.25).

**Table 3:** Heritability, genetic correlation, and phenotypic correlation between working and medium trot (SE in brackets).

	Heritability Working	Heritability Medium	Genetic correlation	Phenotypic correlation
Velocity (V)	0.03 (0.04)	0.10 (0.07)	0.98 (0.58)	0.42 (0.02)
Stride frequency (SF)	0.52 (0.10)	0.40 (0.09)	0.99 (0.03)	0.66 (0.02)
Regularity (R)	0.02 (0.03)	0.07 (0.05)	0.36 (0.80)	0.25 (0.03)
Symmetry (S)	0.06 (0.06)	0.00 (0.04)	-	0.08 (0.03)
Dorsoventral displacement (DVD)	0.51 (0.09)	0.42 (0.09)	0.92 (0.05)	0.63 (0.02)
Dorsoventral activity (DVA)	0.32 (0.08)	0.34 (0.09)	0.97 (0.04)	0.70 (0.01)
Longitudinal activity (LGA)	0.37 (0.09)	0.35 (0.09)	0.97 (0.03)	0.79 (0.01)
Lateral activity (LTA)	0.21 (0.07)	0.12 (0.06)	1.00 (0.09)	0.65 (0.02)

**Table 4:** Heritability, genetic correlation, and phenotypic correlation between working and medium canter (SE in brackets).

	Heritability Working	Heritability Medium	Genetic correlation	Phenotypic correlation
Velocity (V)	0.18 (0.07)	0.08 (0.06)	0.90 (0.28)	0.42 (0.02)
Stride frequency (SF)	0.41 (0.09)	0.46 (0.10)	1.00 (0.03)	0.65 (0.02)
Regularity (R)	0.16 (0.07)	0.04 (0.04)	0.98 (0.50)	0.23 (0.03)
Dorsoventral displacement (DVD)	0.52 (0.09)	0.50 (0.09)	1.00 (0.05)	0.50 (0.02)
Dorsoventral activity (DVA)	0.31 (0.08)	0.29 (0.08)	1.00 (0.06)	0.59 (0.02)
Longitudinal activity (LGA)	0.17 (0.07)	0.21 (0.08)	0.86 (0.10)	0.66 (0.02)
Lateral activity (LTA)	0.12 (0.07)	0.01 (0.05)	-	0.50 (0.02)

For the remainder of the analyses, we considered the trot (T) as a single gait, with two repeated measurements. The same strategy was applied for the canter (C).

### How can we summarize the main characteristics of the walk, trot, and canter by accelerometry?

**Fig 1:**
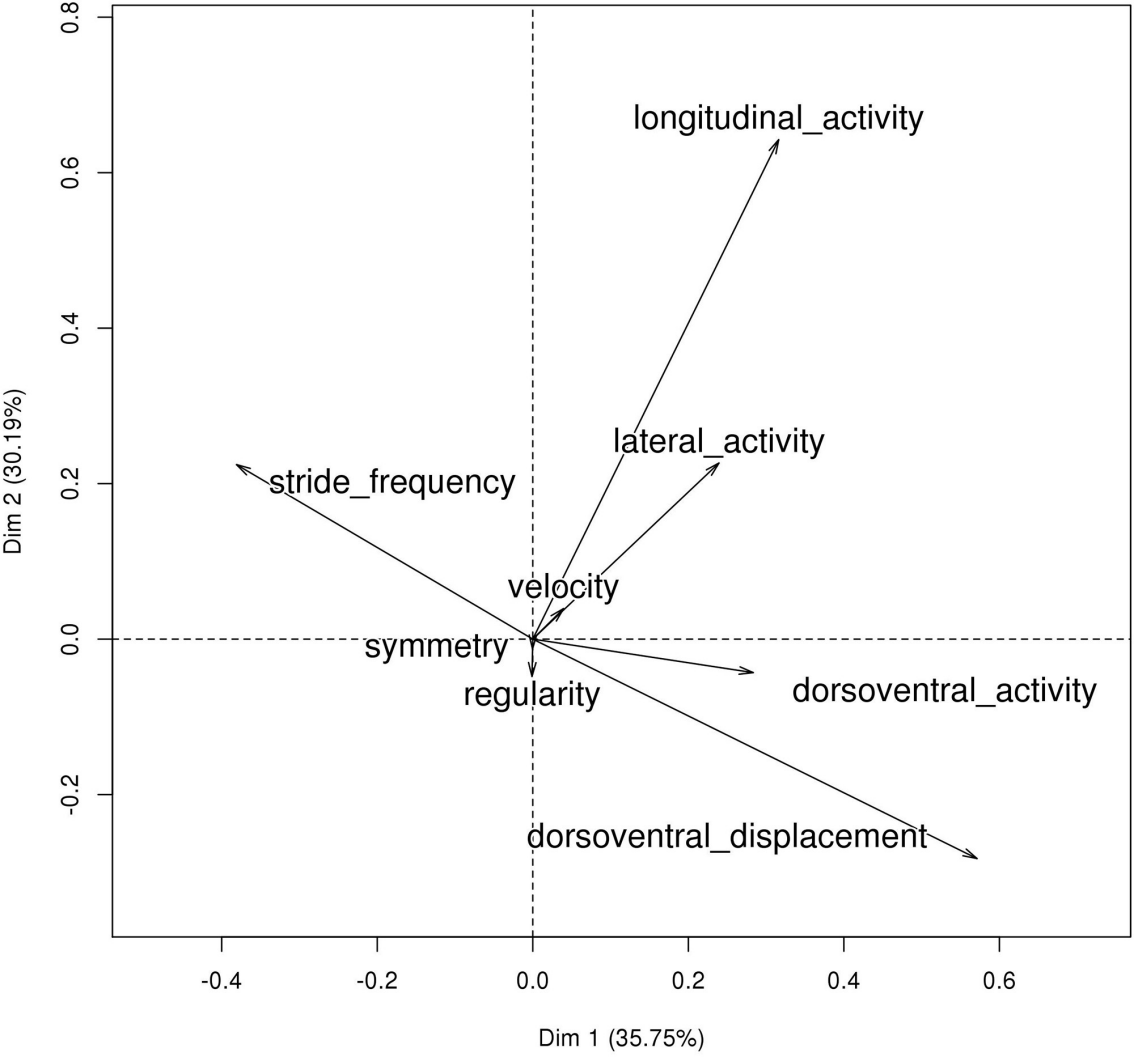
Principal component analysis of trot.

**Fig 2:**
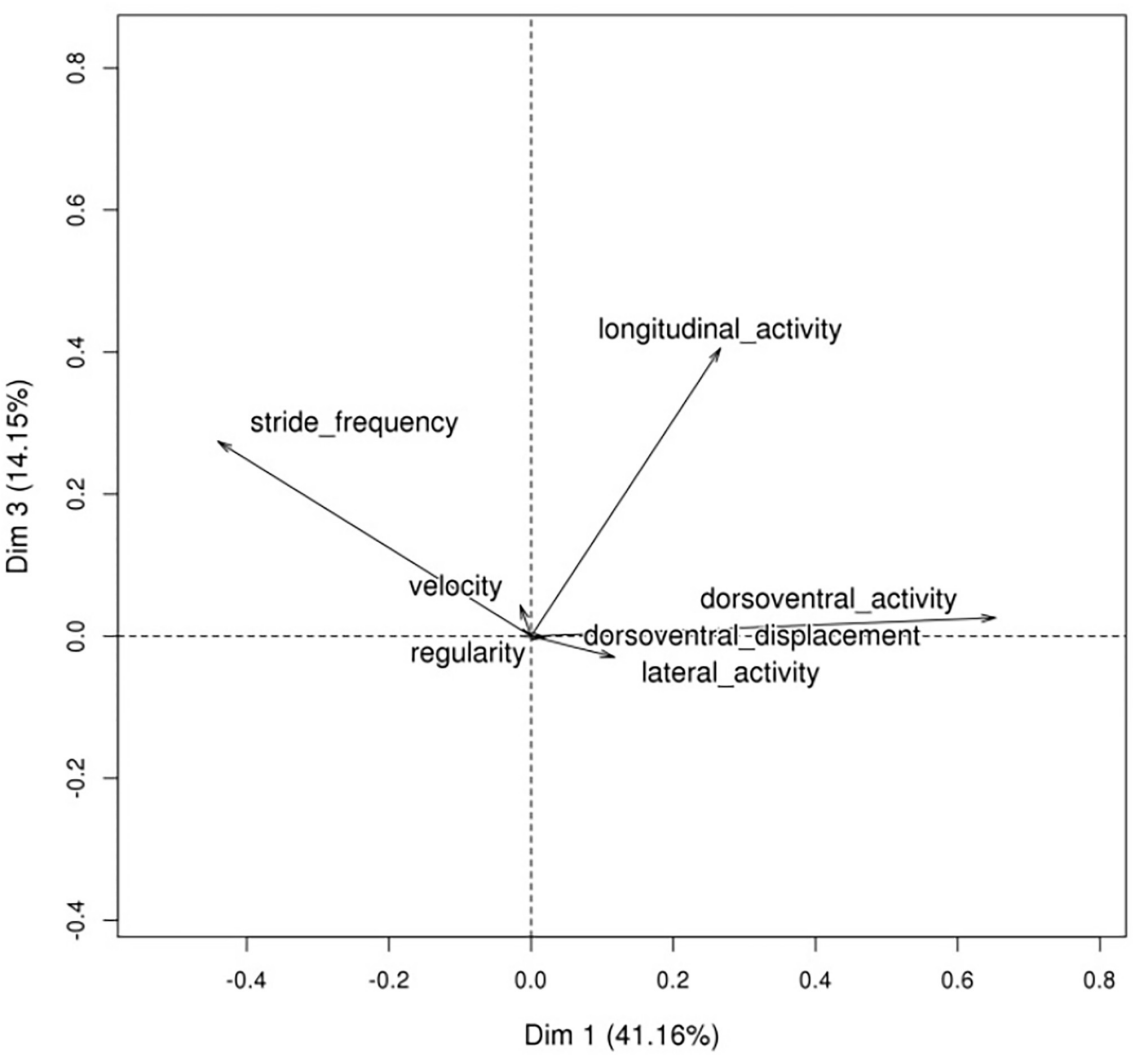
Principal component analysis of canter.

**Fig 3:**
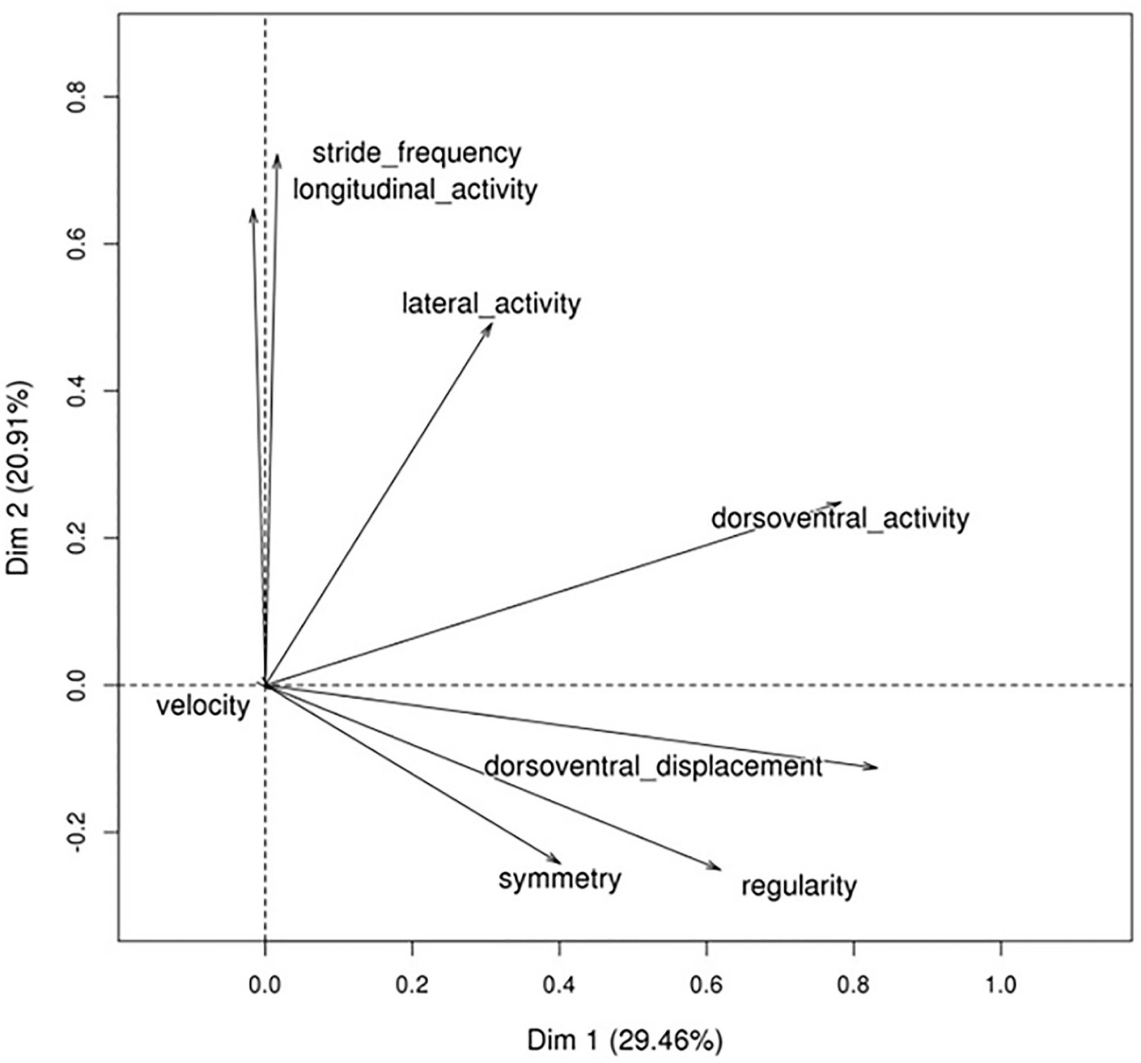
Principal component analysis of walk.

The principal component analyses of the horse effects on the eight measurements at each gait (seven for canter) are illustrated in Figs 1–3 and eigenvectors are given in S1 Table. Trot and canter had similar patterns. The first component was the opposition between DVD and SF (a low stride frequency correlated with a high vertical displacement). The second component (for the trot) and the third (for the canter) was the extent of LGA associated with LTA and SF for trot, or with SF only for canter. The last component considered (third for trot, and second for canter) was LTA (slight association with DVD and SF, and negatively with LGA for trot). These three first principal components represented 82% of the variance for the trot and 89% for the canter. For walk, the first component was DVA and DVD associated with R and S but not SF, which was represented in the second component associated with LGA. The third was SF slightly opposed to R and DVA. These three first PCs represented 64% of the variance. In the rest of the analyses, the PCs will be named according to their rank (1, 2, 3), the gait (T, C, W), and the most important variable (e.g., the first component for trot is PCT1_DVD).

### What is the influence of the height at withers on the gait characteristics measured by accelerometry?

**Fig 4:**
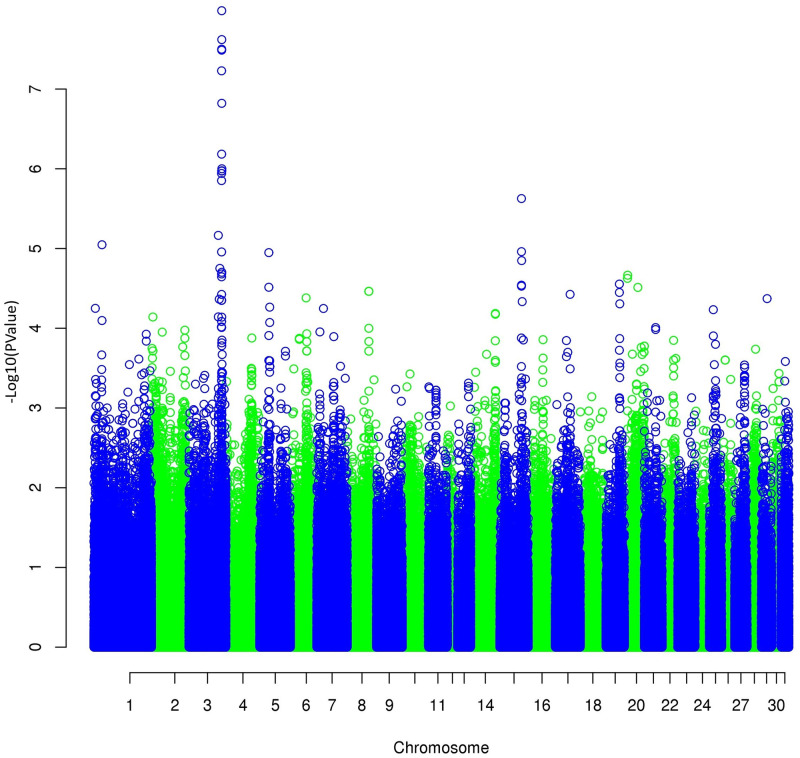
Manhattan plot of the GWAS for the first principal component of trot (PCT1_DVD) involving dorsoventral displacement and stride frequency.

**Fig 5:**
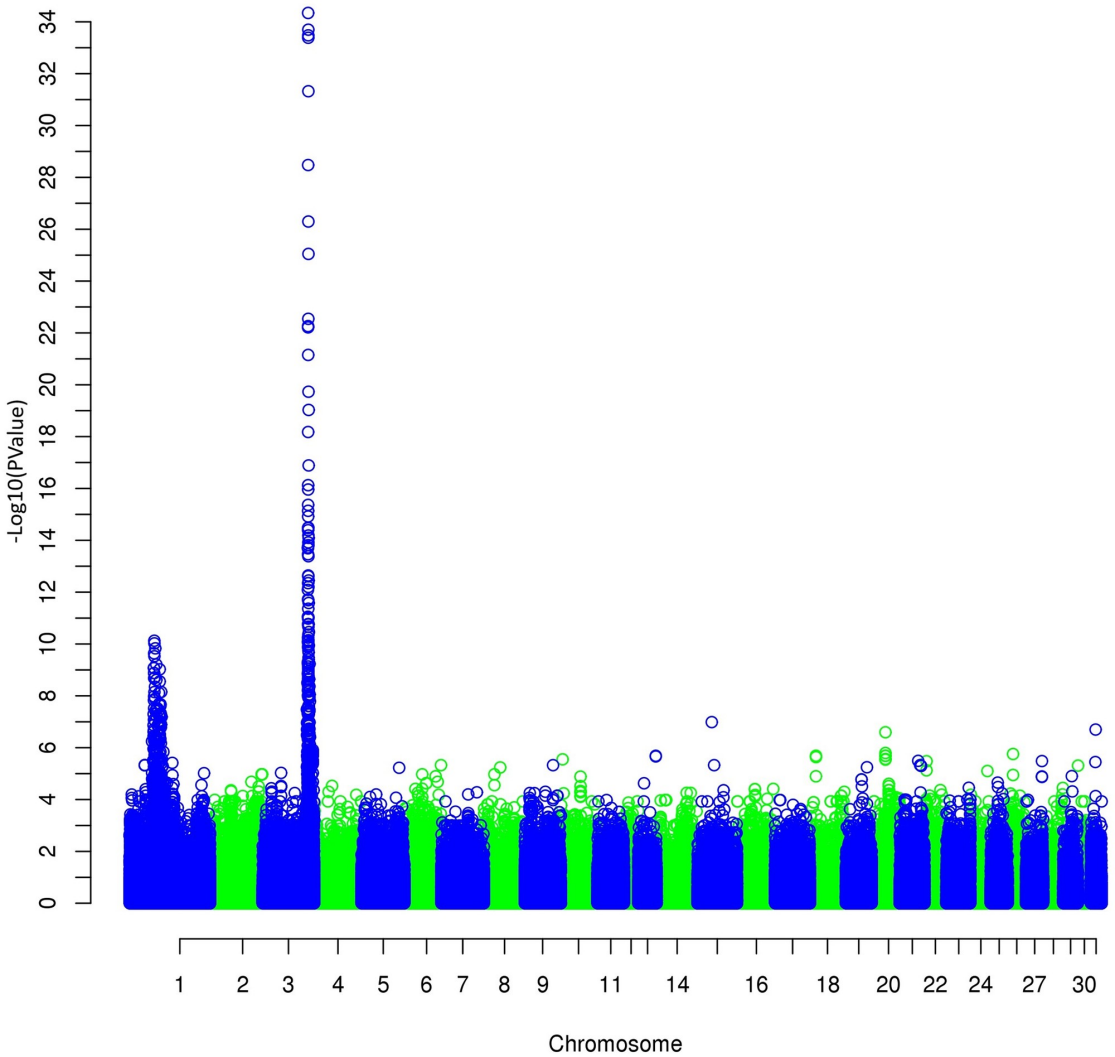
Manhattan plot of the GWAS for the height at withers.

**Fig 6:**
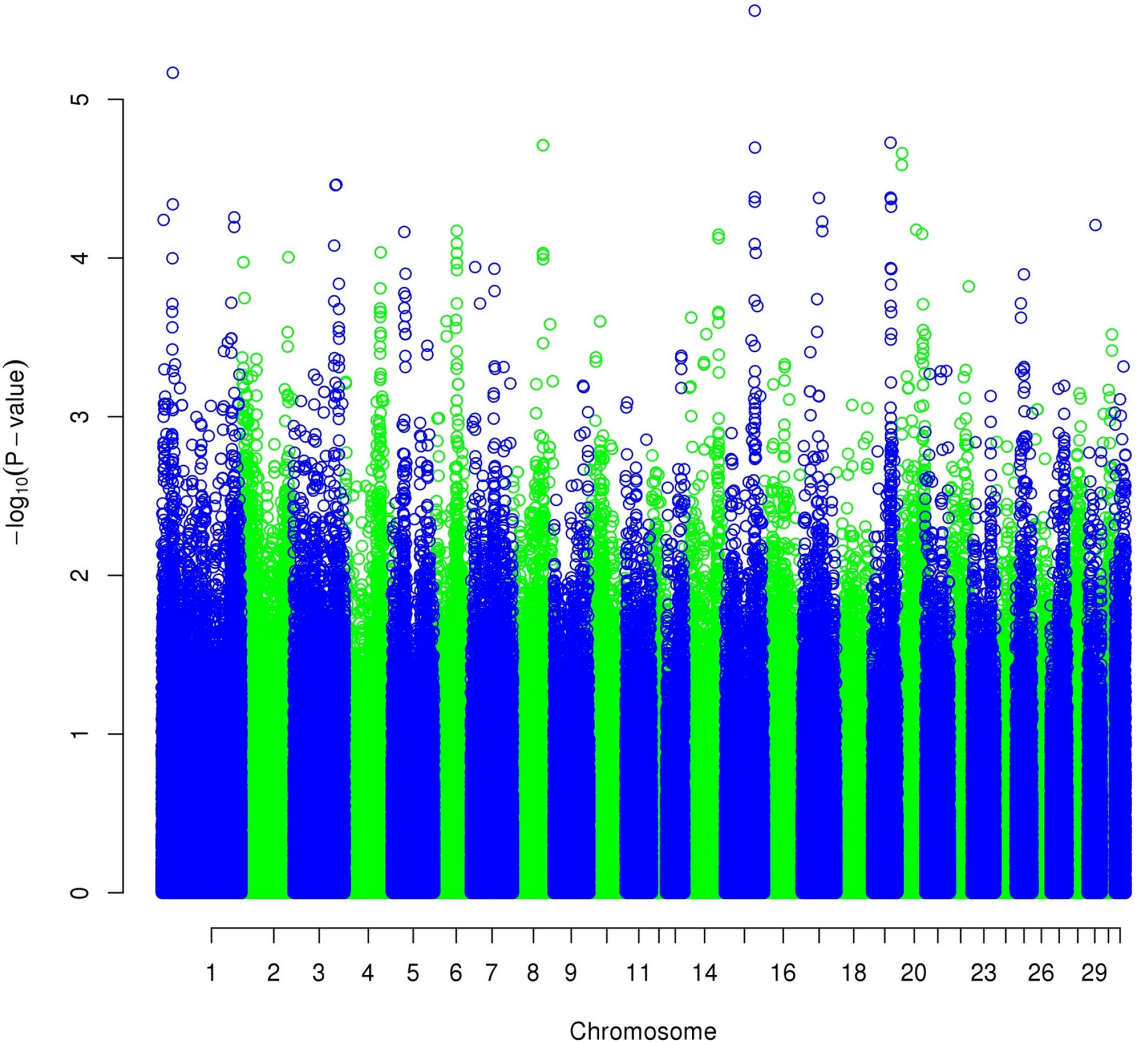
Manhattan plot of the GWAS for PCT1_DVD corrected for the height at withers.

The results of the GWAS (Fig 4) on the first principal component for trot revealed a highly significant result (*p* = from 1.0 x 10^−6^ to 1.0 x 10^−8^) for the region on chromosome 3 (3:105,152,868 to 3:106,128,177 on EquCab2.0) close to the one linked to the height at withers already found by Signer-Hasler et al. [24], Frischknecht et al. [25], and Makvandi-Nejad et al. [26]. We, therefore, also performed a GWAS on the height at withers (Fig 5), and we found exactly the same region, with an even much lower *p* = 4 x 10^−35^. However, the objective of measuring gait characteristics was not to distinguish tall and small horses. Thus, we decided to differentiate the two traits with a structured equation model: gait components were analyzed using the height effect, and height was added to the genetic analysis as a supplementary trait. No other significant regions were found on gait characteristics after height correction (Fig 6).

### Genetic analysis of gaits measured by accelerometry and the height at withers

Aging (from 4 to 5 years old) had a negative effect on all of the variables, albeit non-significant for walk. Females had a lower PCT1_DVD and PCC1_DVD, and a higher PCW2_SF, meaning a higher stride frequency in each case (even when corrected for their lower height). They had a higher PCC3_LGA. Geldings were not significantly different from stallions, except for their PCT1_DVD and PCT2_LGA, which were lower than in stallions but higher than in mares for DVD. The event effect was substantial: the range was often higher than one phenotypic standard deviation unit.

The effect of velocity on PCs was described with the estimates of effect of the type of gaits (medium versus working), knowing that medium gaits had a higher velocity than working gaits in average, and with the linear regression coefficients for velocity within each type of gait (Table 5). The linear regression coefficients for height at withers were also presented in Table 5. All of the variables increased with velocity. PCT1_DVD and PCC1_DVD increased with height, but PCT2_LGA and PCC3_LGA decreased with height, and height had a minor influence on PC_LTA and all PCW. Estimates of the effects linked to velocity and height at withers on initial variables describing gaits were available in S2 Table.

**Table 5:** Effect of the type of gait (medium versus working), regression coefficient on velocity within type of gait and height at withers for the nine principal components of gaits, all expressed in phenotypic standard deviation units.

	Medium versus Working (reference)	Velocity regression coefficient within working	Velocity regression coefficient within medium	Height at withers
PCT1_DVD	0.288 (0.018)	0.108 (0.017)	0.126 (0.017)	0.216 (0.022)
PCT2_LGA	1.006 (0.017)	0.288 (0.015)	0.232 (0.015)	-0.217 (0.020)
PCT3_LTA	1.256 (0.018)	0.355 (0.016)	0.271 (0.016)	-0.081 (0.017)
PCC1_DVD	0.877 (0.020)	0.225 (0.019)	0.184 (0.018)	0.137 (0.019)
PCC2_LTA	0.789 (0.024)	0.216 (0.022)	0.296 (0.022)	0.044 (0.021)
PCC3_LGA	1.085 (0.018)	0.299 (0.016)	0.373 (0.016)	-0.176 (0.016)
		Velocity regression coefficient		
PCW1_DVD		0.363 (0.030)		0.013 (0.025)
PCW2_SF		0.438 (0.028)		-0.050 (0.024)
PCW3_S		0.139 (0.033)		-0.044 (0.027)

Names of the variables: PCx = Principal Component, x = Rank of the PC, T = Trot, C = Canter, W = walk, DVD = Dorsoventral displacement, LGA = Longitudinal activity, LTA = Lateral activity, SF = Stride frequency, S = Symmetry.

The results of the 10 multiple traits analysis are shown in Table 6. For trot and canter, the heritabilities were higher for PC_DVD (0.53, 0.41) than PC_LGA (0.33, 0.19) and low for PC_LTA. They were higher for trot than canter. The heritabilities for walk were low (≤ 0.16). The heritability for height at withers was moderate (0.33). No significant correlation between traits for the same gait was found. The genetic correlations for similar traits (PCT1 and PCC1 on the one hand, PCT2 and PCC3 on the other hand) between canter and trot were high (0.56, 0.73) except for LTA (PCT3 and PCC2). The PCW2_SF, which included stride frequency and activities, were correlated to a similar PC at trot and canter (PCT2_LGA and PCC3_LGA). It was also negatively correlated to PCT1_DVD, which included a negative coefficient for the SF variable. Despite the regression already included with height, a positive genetic correlation between height and PCT2_LGA and PCT3_LTA was found.

**Table 6:** Heritability (diagonal), genetic correlation (above the diagonal), and phenotypic correlation (below the diagonal) between the principal component traits of gaits and height at withers.

	PCT1_DVD	PCT2_LGA	PCT3_LTA	PCC1_DVD	PCC2_LTA	PCC3_LGA	PCW1_DVD	PCW2_SF	PCW3_S	Height
PCT1_DVD	0.53 (0.06)	-0.18 (0.15)	-0.31 (0.21)	0.56 (0.10)	0.00 (0.26)	-0.23 (0.18)	0.33 (0.20)	-0.54 (0.19)	0.13 (0.39)	0.00 (0.09)
PCT2_LGA	-0.06 (0.02)	0.33 (0.04)	0.07 (0.24)	-0.15 (0.16)	-0.37 (0.32)	0.73 (0.11)	-0.27 (0.23)	0.49 (0.19)	0.43 (0.51)	0.21 (0.11)
PCT3_LTA	0.02 (0.02)	0.16 (0.02)	0.11 (0.04)	-0.10 (0.23)	0.34 (0.42)	0.20 (0.28)	-0.13 (0.35)	0.52 (0.28)	0.16 (0.64)	0.36 (0.18)
PCC1_DVD	0.37 (0.02)	0.02 (0.02)	0.04 (0.02)	0.41 (0.06)	0.27 (0.26)	-0.31 (-0.19)	0.25 (0.22)	-0.11 (0.21)	0.04 (0.42)	0.08 (010)
PCC2_LTA	0.00 (0.02)	0.20 (0.01)	0.08 (0.02)	0.28 (0.02)	0.07 (0.03)	-0.41 (0.41)	-0.20 (0.42)	-0.10 (0.38)	0.14 (0.81)	-0.06 (0.21)
PCC3_LGA	-0.01 (0.02)	0.43 (0.02)	0.07 (0.02)	0.19 (0.02)	0.31 (0.02)	0.19 (0.05)	-0.24 (0.27)	0.48 (0.22)	0.60 (0.62)	0.00 (0.13)
PCW1_DVD	0.11 (0.02)	0.04 (0.02)	-0.08 (0.02)	0.09 (0.02)	0.06 (0.02)	0.00 (0.02)	0.16 (0.07)	-0.45 (0.30)	-0.68 (0.69)	0.22 (0.17)
PCW2_SF	-0.04 (0.02)	0.21 (0.02)	0.19 (0.02)	0.05 (0.02)	0.16 (0.02)	0.23 (0.02)	0.00 (0.03)	0.15 (0.06)	0.26 (0.60)	-0.21 (0.16)
PCW3_S	-0.02 (0.02)	0.09 (0.02)	0.04 (0.02)	0.00 (0.02)	0.03 (0.02)	0.06 (0.02)	0.00 (0.03)	0.00 (0.03)	0.04 (0.06)	-0.07 (0.32)
Height	0.00 (0.03)	0.07 (0.03)	0.07 (0.03)	0.03 (0.03)	-0.01 (0.03)	0.00 (0.03)0	0.05 (0.04)	-0.05 (0.04)	-0.01 (0.04)	0.33 (0.08)

The SE is indicated in brackets and a colored box is used for values significantly different from zero.

Abbreviations for the variables: PCYx_AAA: PC = Principal Component, Y = T for Trot, C for canter, W for Walk, x the rank of the principal component from 1 to 3, AAA = abbreviation for the main measurement involved in the principal component, DVD = Dorsoventral displacement, LGA = Longitudinal activity, LTA = Lateral activity, SF = Stride frequency, S = Symmetry.

### Genetic correlations between accelerometry, jumping competition, and judges’ scores

The genetic correlations are presented in Table 7. The heritability of the annual logarithm sum of the points was 0.31 (SE 0.01) and the annual reproducibility was 0.52 (SE 0.002). Only one genetic correlation between gaits measured by accelerometry and jumping performance was significantly different from zero: -0.19 (SE 0.10) for PCC3_LGA. A high longitudinal activity at canter with a high stride frequency was genetically associated with lower performance. The corresponding phenotypic correlation was -0.02 (SE 0.02). The heritabilities of the gait judges’ scores were moderate: from 0.18 (for canter) to 0.28 (for trot). The genetic correlation between jumping competition performance and judges’ scores for gaits was positive and moderate, 0.17 (SE 0.03) for walk, to quite high 0.45 (SE 0.03) for canter. The genetic correlation between the accelerometry and the judges’ scores showed that judges scored horses with high dorsoventral displacement (low stride frequency for trot and canter) and low lateral and longitudinal activity similarly positively for trot, canter, and walk. The highest correlation was between PCW1_DVD and the walk score: 0.75 (SE 0.19) and PCT1_DVD and the trot score: 0.72 (SE 0.07).

**Table 7:** Heritability (first line), genetic correlations between gaits measured by accelerometry, by judges’ scores, and by jumping competition performance.

		Judge Gait Score		
	Jumping Competition	Trot	Canter	Walk
Heritability	0.30 (0.01)	0.28 (0.01)	0.18 (0.01)	0.19 (0.01)
Accelerometry				
PCT1_DVD	0.02 (0.06)	0.72 (0.07)	0.49 (0.08)	0.37 (0.1)
PCT2_LGA	-0.15 (0.08)	-0.34 (0.11)	-0.25 (0.12)	-0.36 (0.12)
PCT3_LTA	0.00 (0.12)	-0.45 (0.15)	-0.24 (0.18)	-0.49 (0.18)
PCC1_DVD	0.14 (0.07)	0.29 (0.11)	0.29 (0.11)	-0.02 (0.12)
PCC2_LTA	-0.16 (0.16)	-0.48 (0.22)	-0.45 (0.25)	-0.41 (0.25)
PCC3_LGA	-0.19 (0.10)	-0.27 (0.15)	-0.34 (0.15)	-0.53 (0.14)
PCW1_DVD	0.05 (0.13)	0.22 (0.19)	0.36 (0.23)	0.75 (0.19)
PCW2_SF	-0.12 (0.12)	-0.39 (0.18)	-0.21 (0.18)	-0.49 (0.17)
Jumping Competition		0.31 (0.03)	0.45 (0.03)	0.17 (0.03)

### More regarding judges’ scores

The relatively high genetic correlations between gait scores and jumping competition performance, although these are very different traits, prompted us to look at genetic correlations between conformation and gait scores and between conformation and jumping competition performance. High genetic correlations between the scores for conformation traits and gait traits were found (S3 Table): from 0.51 to 0.67 (SE 0.03 to 0.06) for trot and canter and from 0.31 to 0.45 (SE 0.04 to 0.06) for walk. Very high genetic correlations were found between the different conformation traits: from 0.65 to 0.89, average 0.74 (SE 0.01 to 0.04). Irrespective of the conformation trait, the genetic correlation with jumping competition performance was quite high (Table 8): from 0.20 for “chic” to 0.54 for joints (the others were between 0.30 and 0.40).

**Table 8:** Genetic correlation between the judges’ scores and the jumping competition performance: Raw scores and residual scores obtained by regression with the parental jumping competition EBV available when scored (SE in brackets).

	Raw Score	Residual Score
**Conformation**		
Neck	0.24 (0.03)	-0.19 (0.03)
Forehand (shoulder, forearm)	0.39 (0.03)	-0.39 (0.03)
Withers-Back-Loins	0.31 (0.03)	-0.24 (0.03)
Hindquarters (croup, pelvis, thigh)	0.37 (0.03)	-0.34 (0.03)
Joints	0.54 (0.06)	-0.41 (0.06)
Forelimb	0.32 (0.04)	-0.53 (0.03)
Hindlimb	0.28 (0.03)	-0.45 (0.03)
Impression	0.34 (0.03)	-0.11 (0.03)
Chic	0.20 (0.03)	-0.20 (0.03)
**Gaits**		
Trot	0.31 (0.03)	-0.18 (0.03)
Canter	0.45 (0.03)	-0.20 (0.03)
Walk	0.17 (0.03)	-0.38 (0.03)

This homogeneity in the correlations led us to create new variables to characterize the scores: the residuals from the regression of scores as a predicted variable and genetic evaluation for the jumping competition performance from ascendants as known at the time of the scoring. The mean reliability of these genetic evaluations was 0.312. For example, with this reliability and the formula given in the material section, expected genetic correlation for an initial genetic correlation of 0.40 would be 0.28. In practice, the genetic correlation between the jumping competition performance and the residual scores (Table 8) became slightly negative (approximately– 0.20, SE 0.03) or even worse (-0.53 for the forelimbs conformation). It was the same for the scores for the gaits (from -0.18 to -0.38).

No significant genetic correlation was found between the height at withers and the jumping performance (0.13, SE 0.09).

## Discussion

### Analysis of gaits

Accelerometry provided new insight regarding the description of gaits. We found that trot and galop were similarly characterized at medium and at working speed. Three principal components were enough to define each of the gaits: trot, canter, and walk. As expected [27–30] activities (dorsoventral, lateral, and longitudinal) and stride frequency increased from working to medium gait and with the increase of velocity for all gaits, namely trot, canter, and walk. Velocity was not very sensitive to height at withers (slight increase for trot and walk, slight decrease for canter), probably because it is left to the free will of the rider. Therefore, it was not the variation of velocity with height which could explained the influence of height on variables. Activities had different behavior regarding the influence of height at withers: longitudinal activity decreased, lateral activity increased and dorsoventral activity was independent from height at withers for the three gaits. Stride frequency decreased with height at withers for all gaits: holding speed constant, horses with longer limbs have a longer stride length, i.e. a lower stride frequency than smaller horses. Dorsoventral displacement was obtained by double integration of dorso-ventral acceleration signal. For trot, it decreased with velocity and increased for walk and canter. This behavior must be directly linked to the mechanism of gait. All dorsoventral displacement increased with height at withers. Regularity and symmetry decreased with an increase of velocity at trot and canter from working to medium gait and within each type of gait (negative regression coefficient for velocity). For walk, it was the opposite (positive regression coefficient for velocity).

The measurement retained to describe gaits should be understood as velocity-corrected variables, not raw measurements. Activities mean the amount of acceleration and deceleration along the axis concerned developed to maintain the same velocity. Genomic analysis of the gaits measured by accelerometry revealed no major QTLs, except the one related to height. The power of GWAS was not very high with only 702 genotyped horses, therefore only very major genes as the one related to height could have been found. A major gene on gaits, a mutation in DMRT3 gene, was found for pace and trot in gaited breeds and breeds for trotting races [31, 32]. But the frequency of allele A at DMRT3, linked to the ability to pace, was two low in our sample (0.9%) to be studied. Gait traits as measured by accelerometers in an already selected population for jumping, seemed to be polygenic traits. However, the gait traits were very heritable, except for regularity and symmetry We measured the gaits of supposedly healthy horses, with less variation in the parameters of regularity and symmetry than horses usually used to identify lameness by accelerometers. Imbalance in gaits was studied by Becker et al. [33] for mares in breeding shows, and they also found low heritabilities for this trait (from 0.03 to 0.12). Trot and canter had the same genetic properties. The PCs for trot and canter were the linear combination of the same variables and there were high genetic correlations between the PCs which had the same meaning: PCT1_DVD and PCC1_DVD (0.56), PCT2_LGA and PCC3_LGA (0.73). Walk was somewhat different because PCs did not rely to the same group of variables than for trot or canter. However, there were still high correlations for PCs including the stride frequency at all gaits: PCT1_DVD and PCW2_SF (-0.54), PCW2_SF and PCC3_LGA (0.48). The heritabilities of the principal components of gaits, even after the inclusion of height at withers in the model, remained high: a selection of specific gaits, independent from the height at withers, is possible.

Estimates of heritabilities based on kinematic analysis (video or accelerometers) of gaits can be found in a handful of publications [34–37] but the results are harder to compare because the methodology and conditions under which the horses were measured is widely different, especially between optical motion capture and accelerometers.

### Relationship with jumping competition performance

The genetic relationships between gaits measured by accelerometers and jumping performance in competition were weak. Only one principal component for canter was significantly genetically linked to performance in jumping competition: a lower longitudinal activity at canter correlated with a slightly higher performance in competition (r_g_ = -0.19, SE 0.10). At first glance, it is surprising that lower activity gave better results in jumping competition. Longitudinal activity is linked to deceleration and acceleration along the longitudinal axis. After correction for velocity, longitudinal activity is therefore linked to the amount deceleration and acceleration necessary to maintain a similar velocity. If these acceleration or deceleration increase without increase of velocity, it may represent loss of efficacity in the movement of canter. This can explain the negative correlation. Other gait characteristics were mostly genetically independent of the jumping performance.

This was not the case for gaits measured by judges’ scores during breeding shows. The genetic correlation between the score for walk and jumping performance was low (0.17, SE 0.03) but relatively high for trot (0.31, SE 0.03) and especially high for canter (0.45, SE 0.03). At first glance, it can be interpreted as an accurate assessment of gaits, specifically for jumping ability and not only for dressage, and it is hence able to predict the potential for a good jumping performance in competition. This is in accordance with the literature. In Sweden, Wallin et al. [38], and then more recently Viklund et al. [39], found a genetic correlation of approximately zero between the score for walk in RHQT (Riding Horse Quality Tests of young horses at 4 years of age) and show jumping results, but 0.18 (0.06) for trot and 0.39 (0.06) for canter. In the Netherlands, Ducro, Koenen [11], and then more recently Rovere, Ducro [5], found a genetic correlation between show jumping and scores obtained in studbook entry inspections of approximately zero for walk, up to 0.11 (0.03) for trot, and 0.41 (0.05) for canter according to the descriptive trait used (length, elasticity, impulsion, or balance). Perhaps the judges saw more qualities and information when analyzing canter than can be measured by accelerometers. The description of canter involved, for example, impulsion, balance, and carriage, which are less readily related to physical measurements made by accelerometers than stride length [5, 11]. But these good achievement of the judges’ scores of gaits for the relationship with jumping competition, which were not largely confirmed by accelerometers measurements, were put into perspective when we came to the realization that all of the scores for conformation were in the same magnitude of correlation and very well correlated between them. The genetic correlation of the conformation scores and jumping in competition varied from 0.20 (0.03) for “chic” to 0.54 (0.06) for joins (average 0.33). The genetic correlation between the scores varied from 0.52 (0.04) between joints and neck to 0.93 (0.02) between joins and hindquarters (average 0.74). The same was observed in [39], with a genetic correlation with show jumping competition from 0.26 (0.06) (head, neck, body) to 0.34 (0.06) (type) and in [5], for example 0.23 with the conformation score. The scores, irrespective of the item, reflected the “good impression” given by the horse. This “good impression” may be influenced by the knowledge of the breeder or the genealogy of the horse. Genealogy interpreted by judges may be related to breeding values published for the sire and the mare of the horse, or, as in France, by the EBV for the horse based on the mean of the parents, which is available on the internet (https://www.ifce.fr/). This is why we also calculated the genetic correlation between the jumping performance and the residual of scores adjusted for the EBV calculated at the age at which the breeding show took place so before any of their own performances in jumping competition. We found negative genetic correlations, even for the gait scores. The scores did not add supplementary information genetically positively related to jumping performance over the parental EBV.

Nevertheless, genetic correlation between the gait scores and the accelerometric variables helped to gain an understanding of the assessment criteria. A good score for trot and canter was genetically linked to high dorsoventral displacement, low stride frequency, and low longitudinal and lateral activities. For walk, the link was with high dorsoventral activities and displacement, regularity, and low stride frequency and longitudinal activity. These genetic correlations were high up to 0.75. And coherence was found despite everything in the detailed genetic correlation between the gait scores and the accelerometric variables for the relationship with jumping competition performance. The genetic correlation between jumping performance and PCC3_LGA (-0.19) was confirmed by the genetic correlation between the canter score and PCC3_LGA (-0.34) and the canter score and jumping performance (0.45).

## Conclusion

The main conclusion is the genetic independence between gaits characteristics and jumping performance. In consequence, selection on gaits traits, whatever they are, will no improve or deteriorate the jumping ability. The only exception is the following. Low longitudinal activity and a low stride frequency at canter for a given velocity are slightly favorable traits for jumping competition performance. Distinct selection for height, irrespective of the goal (increase or decrease), is possible when the genetic evaluation for gaits is based on a structural equation model, as was done here. No other QTLs were found that could help with selection, other than the one linked to the height at wither, which naturally led to a lower stride frequency and a higher dorsoventral displacement at trot and at canter.

Judges’ scores for gaits were clearly related to specific gait variables quantifiable by accelerometers. But they were genetically correlated much more efficiently to performance in jumping competition than the accelerometric variables. This may be because judges see more qualities and characteristics in gaits than can be produced with accelerometers, but perhaps also because they know the genealogy of the horse and thus their parental EBV for jumping competition. The contribution of information regarding gaits produced by the judges who are cognizant of this EBV is not positively genetically correlated to jumping competition performance.

Measuring horses with an accelerometer will not be helpful for selecting horses for jumping performance. However, it may be useful if the performance in competition is not the only goal of the breed and if gait traits are a goal themselves. In that case, accelerometers provide objective, easy to record and heritable traits to select.

## Supporting information

S1 Table Eigenvectors of the principal component analysis of accelerometric measurement of gaits. (XLSX)

S2 Table Effect of velocity and height at withers on the accelerometric variables. (XLSX)

S3 Table Genetic correlation between conformation and gait scores. (XLSX)

S1 File Distribution of points allocated to event and rank to calculate the annual jumping performance. (PDF)

S2 File Genetic correlation between two traits when one trait is the residual of the regression of the trait with the parental breeding value of the other trait as the dependent variable. (PDF)
