# Supplementary material for: Genetic consistency between gait analysis by accelerometry and evaluation scores at breeding shows for the selection of jumping competition horses
Source: PLoS One. 2020 Dec 16;15(12):e0244064. doi: 10.1371/journal.pone.0244064 (PMC7743953; doi:10.1371/journal.pone.0244064)

Points allocated to the event according to height of obstacle for a "Grand Prix" event for national and international competitions.

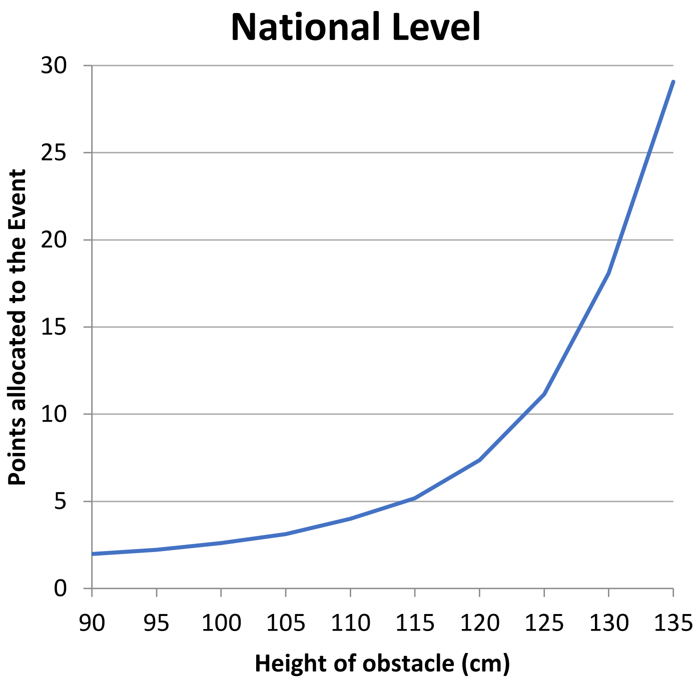

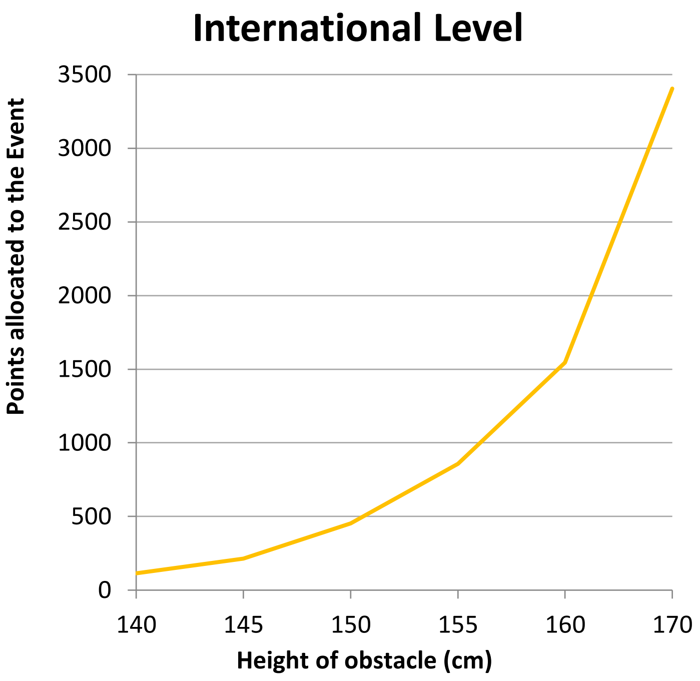

Points allocated according to the rank of the horse with different cases of number of starters.

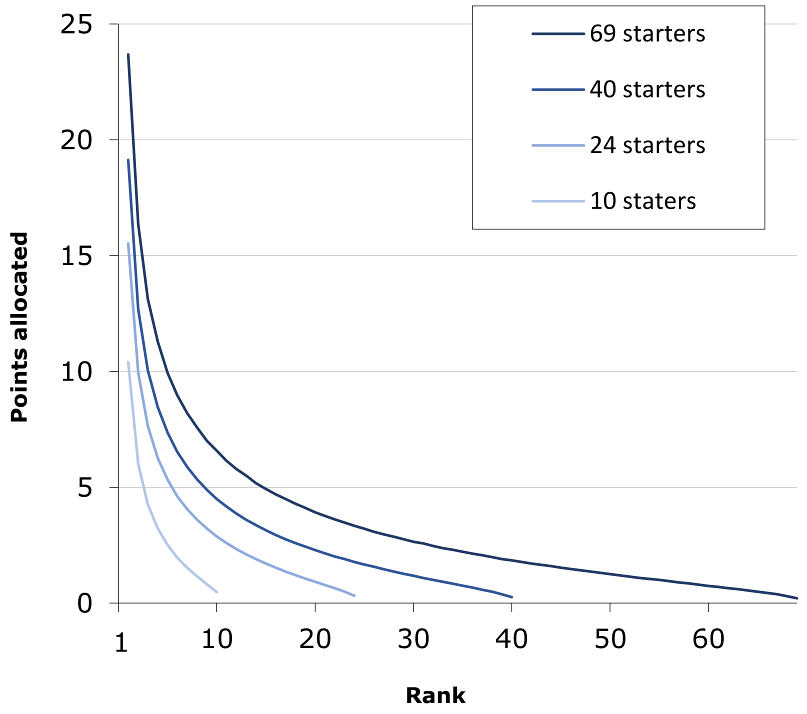

Supplement: S1 File — (PDF) [file pone.0244064.s004.pdf]
