# Supplementary material for: Genetic consistency between gait analysis by accelerometry and evaluation scores at breeding shows for the selection of jumping competition horses
Source: PLoS One. 2020 Dec 16;15(12):e0244064. doi: 10.1371/journal.pone.0244064 (PMC7743953; doi:10.1371/journal.pone.0244064)
Supplement: S2 File — (PDF) [file pone.0244064.s005.pdf]

Consider two traits

$$y_1 = \mu_1 + a_1 + e_1$$

$$y_2 = \mu_2 + a_2 + e_2$$

For simplicity the phenotypic standard deviation was set to 1.

Let $\hat{a}_1^0$ be an estimate of a involving only relatives from parental information.

Performed a linear regression:

$$y_2 = b\hat{a}_1^0 + \varepsilon$$

The regression coefficient was

$$b = \frac{cov(\hat{a}_1^0, y_2)}{V(\hat{a}_1^0)} = \frac{CD_0 cov(a_1, a_2)}{CD_0 V(a_1)} = r_a\sqrt{\frac{h_2^2}{h_1^2}}$$

Because the own performance was not used to compute $\hat{a}_1^0$

Then the new score y_2^* was:

$$y_2^* = y_2 - r_a\sqrt{\frac{h_2^2}{h_1^2}}\hat{a}_1^0$$

The variance of these new performance $y_2^*$was

$$V\left(y_2 - r_a\sqrt{\frac{h_2^2}{h_1^2}}\hat{a}_1^0\right) = 1 - r_a^2\frac{h_2^2}{h_1^2}CD_0h_1^2 = 1 - r_a^2CD_0h_2^2$$

The reduction of variance does not affect the residual variance; hence, the new heritability was:

$$\frac{1 - r_a^2CD_0h_2^2 - (1 - h_2^2)}{1 - r_a^2CD_0h_2^2} = \frac{h_2^2(1 - r_a^2CD_0)}{1 - r_a^2CD_0h_2^2}$$

$$h_2^{2*} = \frac{h_2^2(1 - r_a^2CD_0)}{1 - r_a^2CD_0h_2^2}$$

The covariance between y_1 and y_2^* was:

$$cov\left(y_1, y_2 - r_a\sqrt{\frac{h_2^2}{h_1^2}}\hat{a}_1^0\right) = cov(a_1, a_2) + cov(e_1, e_2) - r_a\sqrt{\frac{h_2^2}{h_1^2}}cov(a_1, \hat{a}_1^0)$$

$$= r_a\sqrt{h_1^2h_2^2} - r_a\sqrt{\frac{h_2^2}{h_1^2}}CD_0h_1^2 + cov(e_1, e_2) = r_a\sqrt{h_1^2h_2^2}(1 - CD_0) + cov(e_1, e_2)$$

And because the residual covariance did not change, the genetic covariance was:

$$r_a\sqrt{h_1^2h_2^2}(1 - CD_0)$$

And then the genetic correlation:

$$r_a^* = \frac{r_a\sqrt{h_1^2 h_2^2}(1 - CD_0)}{\sqrt{h_1^2 h_2^2(1 - r_a^2 CD_0)}}$$

$$r_a^* = \frac{r_a(1 - CD_0)}{\sqrt{(1 - r_a^2 CD_0)}}$$
